# Pediatric Speech-Language Pathologists’ Use of Mobile Health Technology: Qualitative Questionnaire Study

**DOI:** 10.2196/13966

**Published:** 2019-09-26

**Authors:** Kelsey Thompson, Emily Zimmerman

**Affiliations:** 1 Communication Sciences & Disorders, Northeastern University Boston, MA United States

**Keywords:** mHealth, speech-language pathology, surveys, assessment, pediatric, treatment, technology

## Abstract

**Background:**

While technology use in pediatric therapies is increasing, there is so far no research available focusing on how pediatric speech-language pathologists (SLPs) in the United States use technology.

**Objective:**

This paper sought to determine if, and to what extent, pediatric SLPs are using mobile apps, to determine what purpose they are using them for, and to identify gaps in available technology to provide guidance for future technological development.

**Methods:**

Pediatric SLPs completed an online survey containing five sections: demographics, overall use, use in assessment, use in intervention, barriers, and future directions.

**Results:**

Mobile app use by 485 pediatric SLPs in the clinical setting was analyzed. Most (364/438; 83.1%) pediatric SLPs reported using technology ≤50% of the time in their clinical work, with no differences evident by age group (<35 years and ≥35 years; *P*=.97). Pediatric SLPs are currently using apps for intervention (399/1105; 36.1%), clinical information (241/1105; 21.8%), parent education (151/1105; 13.7%), assessment (132/1105; 12%), client education (108/1105; 9.8%), and other uses (55/1105; 5.0%). Cost (46/135; 34.1%) and lack of an evidence base (36/135; 26.7%) were the most frequently reported barriers. Most SLPs (268/380; 70.7%) desired more technology use, with no difference evident by age group (*P*=.81).

**Conclusions:**

A majority of pediatric SLPs are using mobile apps less than 50% of the time in a pediatric setting and they use them more during intervention compared to assessment. While pediatric SLPs are hesitant to add to their client’s screen time, they would like more apps to be developed that are supported by research and are less expensive. Implications for future research and app development are also discussed.

## Introduction

Mobile health (mHealth) is health information or medical services that are delivered or enhanced through mobile communication and information technology [1]. While its traditional purpose is to support the collection and analysis of health-related information, mHealth also encompasses a growing body of technologies that aim to support both the provider and the patient [2]. For example, applications have been created to enhance clinical decision making and diagnostics, improve treatment, increase access to services, and lower costs [2-6]. On the patient side, mHealth applications have successfully been used for education and behavior changes through direct messaging [7], and to engage patients in generating and recording their own health data [8]. Mobile apps are cost effective, accessible, and convenient, and along with the trend of greater consumer involvement, mHealth is heading in a compelling direction [9,10].

Technology use is rapidly increasing, and not just for adults. Children are interacting with technology at home: more than half of parents have downloaded apps specifically for their children [11,12] and homes with children between 0-8 years old who had a mobile device increased from 52% in 2011 to 98% in 2017 [12]. Tablet ownership specifically increased from 8% in 2011 to 78% in 2017 [12]. Schools are also integrating technology into their classrooms. In 2010, the US Department of Education began a National Education Technology Plan to promote student-centered learning with technology, with the goal of improving student achievement [13]. This plan was updated in 2017 and reported a shift from focusing on whether technology should be used to how it can best be used with equal access [14]. They additionally reported progress in technology use for personalized and adapted learning and assessment, on increased education for teachers on how to use technology to support user outcomes, on more classrooms with high-speed connectivity, on the better design of learning spaces to accommodate technology, and on the lower costs and increased availability of high-quality educational tools [14]. In fact, in 2016, 81% of US PreK-12^th^ grade teachers reported using computers or laptops in their classrooms, 58% reported using interactive whiteboards, and 52% reported using tablets [15].

Despite the obvious growth of mHealth in home, medical, and educational settings, research supporting the outcomes of mHealth in speech-language pathology is just emerging, and research in the United States has been limited. There is a body of research that has examined the use of game-based applications for speech and language disorder intervention [16-21], as well as emerging research on apps for speech and hearing screenings [22,23] and biofeedback [24]. Numerous studies report strong child engagement and motivation with the applications, but improvement in skills and generalization of those skills is limited by methodology (ie, no control group) or is not reported [16-21,24]. In fact, Furlong, Morris, Erickson and Serry (2018) developed a protocol for evidence-based appraisal of mobile apps for speech sound disorders [25], and in a systematic review of the Apple iTunes store and Google Play store for apps for speech disorders they found only a small proportion of applications that would be considered very high quality or therapeutically beneficial [26]. There is early evidence for creating applications that are better informed by a joint team approach that shows promise [27]. App use by speech-language pathologists (SLPs) has been examined in both Slovenia and Portugal, where SLPs have reported a positive perception of technology and personal use, but a limited use for therapy purposes [20,21,28].

However, despite the American-Speech-Language-Hearing Association (ASHA) member newsletter having published numerous articles about mHealth in clinical practice, ranging from promoting specific apps [29-33] to advising clinicians about how to incorporate apps into therapy [30,32,34,35], no evidence is available for how SLPs in the United States are, or are not, utilizing mobile applications.

It is clear that mHealth is a growing trend, with children using mobile and tablet devices at home and school. Furthermore, there is emerging evidence that suggests that how adults interact with children during tablet use plays a strong role in their effectiveness [36-39], and there is limited evidence for its efficacy in speech pathology outside of client motivation [16-21,24]. Thus, it is of utmost importance to understand how educators and clinicians are using mHealth with the children they serve to develop improved, evidence-based technologies and practices. While research on the use of mHealth and barriers to adoption exists in other professions, such as among doctors, nurses, and teachers, the usage of such technologies in the field of speech-language pathology in the United States, specifically pediatrics (birth-18 years old), has not been examined. Filling this gap in knowledge is critical for the implementation of mHealth into a field with numerous mobile application offerings without substantive research on the population utilizing them. Therefore, we aimed to understand if, and to what extent, pediatric SLPs are using mobile apps in clinical practice, barriers to use, and gaps in available technology. The following research survey addresses these main questions:

(1) Do pediatric SLPs use technology in clinical practice and what are the barriers to use;

and

(2) Do pediatric SLPs want more technology available and in which areas?

## Methods

### Development of the Survey

To answer the above research questions*,* an anonymous, open survey was developed using Qualtrics Version 2017 (Qualtrics, Provo, Utah), an online survey platform for academic, administrative, and research purposes. Questions were crafted to cover the aims of the study and allowed for forced choice, select all that apply, side-by-side, and open-ended responses. Survey questions were reviewed by two ASHA-certified SLPs and were judged to have enough face and content validity. Internal consistency was assessed for the primary technology questions, which utilized a Likert Scale, by calculating Cronbach alpha using SPSS version 25 (IBM Corp, Armonk, New York). Results showed that the Cronbach alpha was high (0.85), indicating that the primary technology questions were closely related. Then, a pilot study was deployed to further evaluate the validity and comprehensibility of the questions. SLPs who served as supervisors to graduate students in the university’s SLP program were invited to participate in the initial survey. The original survey encompassed 50 questions across three sections: demographics, technology use for the clinician’s three most frequently seen populations, and a summary section about if they desired more technology and the role of cost. Feedback from the pilot study led to additions to the current survey, including questions about barriers to use, factors that could overcome those barriers, if they desired more technology, and open-ended questions about specific technology they use. Additionally, the original survey was broken down by primary practice area, with different options for how they use technology based on each population. The final survey improved flow, incorporating broad options for technology use, limitations, and future directions, allowing all SLPs to provide answers for all populations and allowing for easier comparison. Incorporating the above changes, the final survey included 37 questions covering five topics: demographic information, overall technology use, technology use in assessment, technology use in intervention, gaps or barriers to use, and future directions. The first two questions were related to inclusion criteria. Next, nine demographic questions were asked related to age, race, and work experience. The following 26 questions related to the main survey topic, technology use. Survey questions were designed by the researchers. The terms technology, mobile apps, and apps are used interchangeably in this manuscript and refer to mHealth, specifically the mobile applications that can be downloaded to a phone or tablet, not the devices themselves. The term technology was chosen in the survey as SLPs are not typically aware of the term mHealth. mHealth related to telepractice was excluded from this survey, as was computer-assisted treatment.

**Figure 1:**
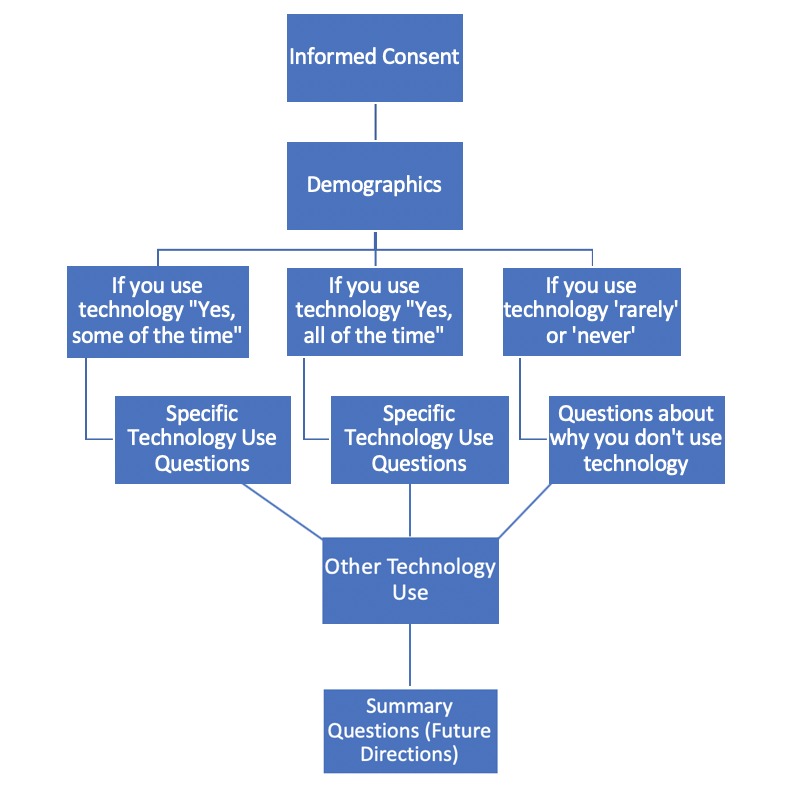
Survey flow diagram.

The final survey questions were not randomized, due to adaptive questioning. Adaptive questioning was used to reduce the number of questions asked when they were not applicable. Due to adaptive questioning, participants saw as few as two screens (if they did not meet the first inclusion criteria) or as many as 11 screens based on their responses (including informed consent). Each screen contained a range of one to six questions per page. Only inclusion criteria questions had to be answered before moving on or completing the survey. Participants were able to revise answers using a back button on the survey. See Figure 1 for survey flow.

### Survey Participants

Participants were recruited using convenience sampling through advertisements on social media and direct emails to pediatric practices from all fifty states. See Multimedia Appendix 1 for the social media announcements and emails.

Investigators posted to pediatric SLP–focused Facebook groups on topics such as pediatric speech therapy, preschool SLPs, early intervention SLPs, and school-based SLPs. The announcement was also posted in research-based groups, such as “SLPs for Evidence Based Practice”. SLPs visit these groups to ask clinical questions, inquire about issues in the field, provide ideas and resources to others, ask questions, present recent research, and occasionally post job openings. Thus, most survey participants were engaged in social media and continuing education in the field. Additionally, private practices were randomly selected using Google searches for “pediatric speech therapy + state name” for all 50 states. The first three listings were emailed the email script (see Multimedia Appendix 1). The survey was available online from November 14, 2017 through May 10, 2018. Inclusion criteria included being ASHA-certified and currently working with a caseload of at least 10 pediatric clients, to ensure the survey population was made up of actively practicing SLPs who would have the opportunity for experience with technology. This survey was approved by the Northeastern University Institutional Review Board before deployment. Informed consent was achieved by having participants read and agree to the Informed Consent before beginning the survey. Informed consent included information about the investigators and their contact information, the purpose of the study, the approximate length of time to complete the survey, and data storage. Participants were cautioned that, due to the nature of the online survey, it is possible they could be identified by IP address or other electronic record associated with their response but that these data were not being actively collected by the investigators. The survey was voluntary, but participants were able to enter a $100 Amazon gift card raffle in exchange for their participation. Personal data was collected in the form of demographic information, which remained anonymous, per the informed consent. Only the investigators had access to the survey portal. Participants were asked to fill out a separate survey with their name and email address in order to enter the raffle, the link to which was listed at the end of the primary survey so that their email was not associated with their response. A total of 621 responses were recorded, of which 518 were ASHA certified. Of the 518 who were ASHA certified, 485 had a caseload of at least 10 pediatric clients, resulting in a study population of 485. Per ASHA’s 2018 year-end counts, 74,764 ASHA-certified SLPs worked with the birth-17 years old age range, thus this survey represents only 0.65% of the population of certified pediatric SLPs.

### Participant Demographics

Participants reported demographic and practice information (see Table 1). Most participants were female (467/485; 96%), white (434/485; 89%), and between the ages of 25-34 (252/485; 52%) or 35-44 (128/485; 26%). Most reported working in a school setting, although all work sites were reported. Except for Hawaii, Nevada, North Dakota, South Dakota, and West Virginia, all remaining states were represented, including the District of Columbia. See Multimedia Appendix 2 for demographic characteristics of the sample.

**Table 1:** Participant demographics (n=485).

Variable		Value, n
**Sex**		
	Female	467
	Male	18
**Age**		
	18-24	14
	25-34	252
	35-44	128
	45-54	59
	55-64	22
	65-74	1
**Ethnicity**		
	White	434
	Black	8
	American Indian or Alaska Native	1
	Asian	17
	Native Hawaiian or Pacific Islander	0
	Other	15
**Years since matriculation with Master's**		
	0-3	120
	4-7	118
	8-11	81
	12+	154
**Work site**		
	Hospital-NICU^a^	5
	Hospital-other inpatient	5
	Hospital-outpatient	40
	Private practice	83
	School	227
	Early intervention	74
	Other	41
**Primary age group working with**		
	Birth to age 3	195
	Preschool (age 3-4)	308
	Early school (age 5-7)	297
	Late elementary (age 8-10)	234
	Middle school (11-13)	134
	High school (14-18)	85

^a^NICU: neonatal intensive care unit.

### Analysis

All entries were analyzed, including incomplete questionnaires. Questionnaires were not monitored for multiple entries or atypical time stamps before analysis. The survey sample was judged to be representative, as it closely aligns with ASHA membership demographics in terms of gender, ethnicity, and work site, so weighting was not utilized. One notable difference is age, which was specifically analyzed using chi square analyses. Age was divided into two categories of near equal population size: age 18-34 years (n=254) and 35 years and older (n=201). The average time participants spent on the survey was 22 minutes. The average progress (how much of the survey they completed) was 88.2%. Of the 624 surveys opened, 482 were completed, resulting in a completion rate of 77.2%. View and participation rates could not be calculated.

For questions with discrete answers, percentages for each question were calculated automatically using Qualtrics’ analysis of responses. The survey also included open-ended questions about the participants’ barriers to use and desires for future use. Coding and analysis of these responses followed an inductive, iterative process inspired by grounded theory analysis, where responses were analyzed for codes and these codes were then iteratively clustered into higher-level themes [40]. For example, for question 150, participants were asked, “What areas of SLP technology would you like to see improvements?” Responses identified as encompassing codes such as: data, collection, data collection, or documentation were grouped into a theme of ‘data collection’, and this was continued for all codes identified. Following analysis, 26 themes were identified. For all open-response questions, only themes that included at least two respondents were reported. This analysis was completed for all open-response questions.

## Results

### Aim 1: Do Pediatric Speech Language Pathologists Use Technology in Clinical Practice and What are the Barriers to Use?

The first aim of the study was to understand if pediatric SLPs are using technology in clinical practice. A total of 367/457 respondents (80.3%) indicated they use technology all or some of the time. Only 73/457 (16.0%) of the pediatric SLPs reported rarely using technology, and 17/457 (3.7%) reported never using technology. There was not a significant difference between age groups in the use of technology (X^2^_1_=0.221; *P*=.97). See Multimedia Appendix 3 for more information.

Of those who did use technology, 223/438 respondents (50.9%) used it during 0-25% of their clinical practice time, and a total of 364/438 respondents used technology during 50% or less of their clinical practice time. There was not a significant difference in percentage of time used between age groups (X^2^_1_=1.024; *P*=.79).

SLPs who reported using technology were asked how often they used it for assessment and intervention specifically. For assessment, 265/309 (86.0%) used it 0-25% of the time, with no difference by age group again (X^2^_1_=1.676; *P*=.64). For intervention, SLPs used technology more often, with 125/307 (40.7%) reporting using it 0-25% of the time, but 127/307 (41.3%) reported using it 26-50% of the time. Only 39/307 (12.7%) used it 51-75% of the time. and 16/307 (5.2%) used it 75-100% of the time. Again, no significant difference was detected in use during intervention by age (X^2^_1_=0.0817; *P*=.84). Overall, most SLPs did use technology but they did not use it during most of their clinical work.

**Figure 2:**
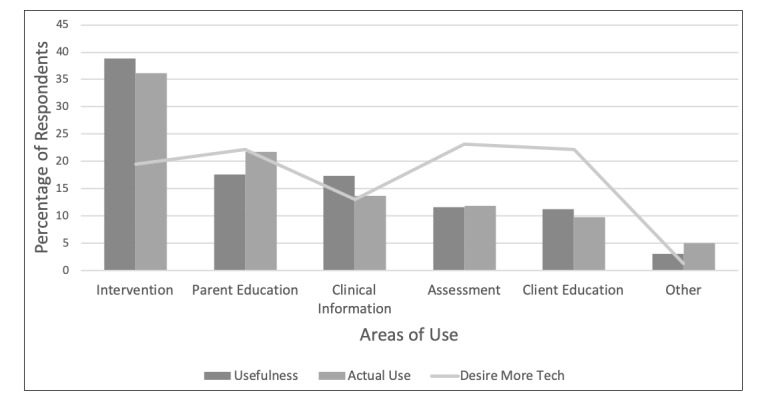
Speech-language pathologists’ ratings of the most useful (dark gray), most used (medium gray) and areas where more technology is desired (light gray) across intervention, parent education, clinical information, assessment, client education and other.

Pediatric SLPs were also asked about what purposes they felt technology was most useful for in a select all that apply type of question. Intervention was most frequently cited (39.0%; 362/929 responses), followed by parent education (17.7%; 164/929), looking up clinical information (ie, developmental norms, treatment techniques) (17.3%;161/929), assessment (11.6%; 108/929), and client education (11.3%; 105/929). Of those who selected other (3.1%; 29/929), a keyword analysis revealed most pediatric SLPs found technology useful for motivation (6/929), augmentative and alternative communication (5/929), and home practice (3/929). Pediatric SLPs were also asked what they are currently using technology for in a select all that apply type of question. Results from a total of 1105 selections were like their ratings for usefulness, and are listed in order of prevalence: intervention (36.1%; 399/1105), clinical information (21.8%; 241/1105), parent education (13.7%; 151/1105), assessment (12.0%; 132/1105), client education (9.8%; 108/1105), and other (5.0%; 55/1105). It is interesting to note that SLPs are currently using apps for what they feel they are most useful for (see Figure 2).

Barriers to technology use was addressed by two questions. The first was a check all that apply type of question, with cost (34.0%; 46/135 responses) and lack of an evidence base (26.7%; 36/135) most frequently reported. Technology not being relevant to their population (13.3%; 18/135) or clinical area (9.6%; 13/135), and not being broad enough to use with a variety of clients (3.7%; 5/135) were not major barriers. Interestingly, 17 pediatric SLPs reported no barriers to using technology (see Figure 2 [SS3] [KT4]). 

An open-ended question about barriers was also presented to discover additional obstacles. Based on a keyword/theme analysis of text responses, 34/131 responses included concerns about not wanting to add to the screen time kids are already getting. Additionally, 11 responses reported anecdotal evidence of children having a tough time transitioning away from screens and 17 responses conveyed feelings that speech and language therapy should be focused on face to face interactions. Other frequently cited concerns included: recommendations for no screen time in early intervention (14/131), not having access to technology (13/131), cost (10/131), focusing on play (10/131), and lack of awareness about which apps to use (6/131).

### Aim 2: Do Pediatric Speech Language Pathologists Want More Technology Available and in Which Areas?

The last section of the survey examined gaps in the availability of technology and future directions. Most pediatric SLPs, 268/380 respondents (70.5%), indicated they wished that there was more technology available “all or some of the time”. This was not affected by age (X^2^_1_=0.974; *P*=.81).

In a select all that apply type of question with 925 total responses, pediatric SLPs desired additional or better technology for: assessment (214/925), parent education (205/925), data recording or viewing (194/925), intervention (180/925), clinical information (120/925), and other (12/925). Pediatric SLPs were also given the opportunity to expand through an open-ended question. Key words and themes extracted from text analysis indicated a strong interest in apps for data collection (11/925), less expensive apps (7/925), evidence-based apps (7/925), language apps (6/925), and customizable apps (4/925). Finally, in a select all that apply type of question, pediatric SLPs indicated they would be more likely to use apps if they were: evidence-based (51/202 responses; 25.3%), cheaper (28/202; 13.9%), targeted a specific skill (27/202; 13.4%), or were endorsed by ASHA (25/202; 12.4%). Less than 10% were interested in apps that were: customizable, broadly applicable, visually enhanced, easier to use, or games that kids were interested in.

## Discussion

### Primary Findings

The purpose of this study was to elucidate the practice patterns of pediatric SLPs in the United States, using mobile technology, to frame the development of future technology for this field. Specifically, we were interested in barriers and desires for future technology. We found that pediatric SLPs were using technology in practice less than half of the time and most frequently for intervention. Pediatric SLPs wanted more evidence for technology use, as they had concerns about screen time and how this may impact development, and they felt that children needed more face to face interactions. They were also concerned about cost. Pediatric SLPs were interested in more technology that focuses on aiding the clinician rather than the child, such as apps for data collection, assessment protocols, and parent education. There was no difference in technology use or desire for future technology based on age group, which is somewhat surprising as research shows younger people are more likely to use mobile technology in general [41], and some research has shown that age is a significant factor in whether teachers use technology [42-45]. However, other, more recent studies suggest that age, or years of experience (typically concurrent with age), are not a significant factor in technology use because young teachers are focused on issues of classroom management and course development, with limited resources left to integrate computers despite their personal experience [46,47].

The recurrent theme across responses was a concern about screen time and the lack of an evidence base for using technology with children. Pediatric SLPs responding to the survey cited concerns about kids getting too much screen time or pointed to the fact that some populations they work with have difficulty transitioning from tablets back to nontablet-based activities, which can hinder the therapy session. Often pediatric SLPs cited the American Academy of Pediatrics’ (AAP) recommendations that screen time should be limited for infants and toddlers, as well as feelings that speech-language pathology treatment should focus on play and face-to-face interactions. While the AAP recommends no screen time for children less than 18 months and limited screen time (1 hour/day), with a focus on educational programming and coviewing for children 18 months to 5 years, the National Association for the Education of Young Children supports the developmentally appropriate and intentional use of technology in early childhood education [48,49]. These conflicting recommendations may challenge pediatric SLPs, particularly when working with the pediatric population where most decisions are made by the parents.

Overall, data shows that how teachers and parents integrate technology with children [36-39], features of the app [36,50], and age [15,51-58] have a strong impact on how effective it is. The available evidence suggests that using technology with children over three years old can support learning and improve motivation when used appropriately and scaffolded by an adult. Given these conclusions promoting the efficacy of technology use, it is critical to understand and address the barriers to technology use for pediatric SLPs. Research on barriers for teachers can help frame the discussion for pediatric SLPs. For example, Ertmer et al [59-61] proposed two types of barriers to technology use: extrinsic (ie, lack of: access, time to learn and use, training, or support) and intrinsic (ie, beliefs, comfort, perceived value) [62]. Other studies have since corroborated these barriers. In this survey, pediatric SLPs cited intrinsic barriers most frequently (beliefs, perceived value, lack of evidence base) as well as extrinsic (cost). Teachers (and presumably pediatric SLPs) have the potential to be positive mediators of the effects of technology on student learning but may not be effectively integrating it into teaching [63,64]. For example, teachers have been found to use technology for homework, communicating with parents, or preparing class materials, but not for direct student teaching [65-67]. While pediatric SLPs in this survey cited intervention as the most used and useful purpose for technology, they cited similarly indirect usage as well, such as using and finding apps most useful for clinical information and parent education, and desiring more technology for indirect activities like data collection. This is not surprising given the limited evidence base for speech- and language-specific applications for use in a therapy setting. However, mobile app use has been shown to increase enjoyment, motivation for, and compliance with therapy in children [16,18,21,23]. Furthermore, proponents of mobile apps for pediatric SLPs suggest apps can help supplement or increase practice time and enhance a family’s engagement with therapy, enhancing the efficiency of traditional therapy [25,68]. There is early evidence for an evidence-based, joint team approach to app development for speech sound disorders that may offer a solution to this problem [27]. It will be important to consider in what contexts apps may be most useful, whether at home for carryover or in the therapy room.

There are a few simple steps that should be taken to increase technology use with SLPs working in a pediatric setting. One is creating and disseminating speech-language therapy specific evidence to support or refute the appropriateness of using technology in speech language pathology assessment and intervention. This will require research into a variety of types of apps and populations, which could take a great deal of time, with limited generalizability for those in the clinical field. This is a broad area that needs to be addressed for a variety of applications, populations, age groups, and settings. Treatment applications that are specifically for use by parents as home carryover and have similarly established efficacy need to be developed.

Applications that offer easy to follow instructions and targets or prompts that the SLP can modify for the family to fit the child’s needs would be beneficial. Another barrier to address is cost; reducing the cost or offering free trials of apps could encourage pediatric SLPs to try apps with their clients, as the majority of pediatric SLPs reported that they are not provided a budget for materials from their place of employment.

Finally, there is an opportunity for development of apps that are adult-facing rather than child-facing, such as apps for data collection, assessment, and parent education. Pediatric SLPs are in a critical position to use technology to enhance a child’s learning and generalization and to educate parents about how to best choose and use apps for their children, as it is evident children are using technology at home regardless of evidence base [12]. Results from this study suggest that extrinsic and intrinsic barriers to adoption are impacting technology use in this clinical field.

### Limitations

There are some limitations to this survey that should be acknowledged. The survey was distributed through email lists and Facebook groups, so participants were already engaged with technology. We were not able to reach pediatric SLPs from all 50 states, and although 45 states were represented, the number of respondents for each state were not proportional to the population. Our participant demographics closely matched those reported by ASHA in terms of gender, ethnicity, and work site, but one notable difference was our participants were younger than most ASHA members [69]. While research shows younger people are generally more likely to use mobile technology, our analyses revealed no difference in technology use or opinions in younger (34 years and under) and older (35 years and older) age groups, consistent with recent research on teachers’ technology use [41,46]. Additionally, we had a primarily white sample (434/485; 89%), which can limit the generalizability of our findings. This is not surprising, however, as ASHA reports 79% of certified speech-language pathologists are white; there is little diversity in the field. Our sample size of 485 was reasonable, but only represents 0.65% of the population of certified pediatric SLPs. Thus, generalizability is limited. Future studies should explore key themes with larger populations and examine the impact work site, years of experience, and location on technology use. While technology was defined at the start of the survey, it is possible that respondents did not read or remember this definition while taking the survey. As a result, some may have considered other specific technologies, like fiberoptic endoscopic evaluation of swallowing or augmentative and alternative communication devices, when answering, which could impact results. Future surveys should offer repeated statements of this definition at the start of each section. Despite these limitations, these results are judged to be representative of the target population, given our study population’s demographics and additional analysis by age group, and offer an early glimpse into the thoughts of pediatric SLPs feelings toward emerging technology. Future studies should more specifically examine subsets of the pediatric SLP populations as well as attempt to reach those not already engaged in social media.

### Conclusions

A majority of pediatric SLPs reported using mobile apps less than 50% of the time in a pediatric setting and used them more during intervention compared to assessment. More research is needed to elucidate the effectiveness of mobile apps for speech and language therapy, to reduce costs, and to develop apps for data collection and parent education to address the barriers to technology adoption in this population.

## Appendix

Multimedia Appendix 1 Social media announcement and email script for study recruitment.

Multimedia Appendix 2 Participant demographics.

Multimedia Appendix 3 Technology use by age group.

## Abbreviations

AAP: American Academy of Pediatrics
ASHA: American-Speech-Language-Hearing Association
mHealth: mobile Health
SLP: speech-language pathologist
