# Supplementary material for: Pediatric Speech-Language Pathologists’ Use of Mobile Health Technology: Qualitative Questionnaire Study
Source: JMIR Rehabil Assist Technol. 2019 Sep 26;6(2):e13966. doi: 10.2196/13966 (PMC6787525; doi:10.2196/13966)
Supplement: Multimedia Appendix 1 [file rehab_v6i2e13966_app1.pdf]

Multimedia Appendix 1: Social Media announcement and email script for study recruitment.

Ver. 2018-1-25

Social Media Text:

Seeking SLPs working in pediatrics to complete a survey about using technology in practice! The survey takes only 10-15 minutes to complete and at the end you can be entered into a drawing for a $100 Amazon gift card. Simply follow this link to participate: https://neu.co1.qualtrics.com/jfe/form/SV_0dG41J6RtHG81g1

Ver. 2018-1-25

Email Script

Dear Clinic/Agency,

The Speech and Neurodevelopment Lab at Northeastern University is surveying pediatric speech-language pathologists about their use of technology (mobile applications) in clinical practice.

We are asking you to share this survey with your speech-language pathologists so we can learn more about the technology SLPs are using today. The survey should take no more than 10-15 minutes for them to complete. At the end of the survey, participants will be able to enter a drawing for a $100 Amazon gift card.

Please share the link here for SLPs to access the survey:
https://neu.co1.qualtrics.com/jfe/form/SV_0dG41J6RtHG81g1

We appreciate you sharing this and your participation. If you have any questions or concerns please feel free to contact myself, the research assistant,
at Thompson.kels@husky.neu.edu or the principal investigator, Dr. Emily Zimmerman,
at e.zimmerman@neu.edu

Thank you,

Dr. Emily Zimmerman
Speech and Neurodevelopment Lab
Northeastern University

Kelsey Thompson, MS, CCC-SLP
Speech and Neurodevelopment Lab
Northeastern University

This study has been approved by the Northeastern University Institutional Review Board (#18-01-21)
