# Supplementary material for: Pediatric Speech-Language Pathologists’ Use of Mobile Health Technology: Qualitative Questionnaire Study
Source: JMIR Rehabil Assist Technol. 2019 Sep 26;6(2):e13966. doi: 10.2196/13966 (PMC6787525; doi:10.2196/13966)
Supplement: Multimedia Appendix 2 [file rehab_v6i2e13966_app2.pdf]

Multimedia Appendix 2: Participant demographics

**Variable**		**Characteristic of sample (*n* =485)**
**Sex - female**		467
**Age**		
	18-24	14
	25-34	252
	35-44	128
	45-54	59
	55-64	22
	65-74	1
**Ethnicity**		
	White	434
	Black or African American	8
	American Indian or Alaska Native	1
	Asian	17
	Native Hawaiian or Pacific Islander	0
	Other	15
**Years since matriculation with Master's**		
	0-3	120
	4-7	118
	8-11	81
	12+	154
**Work Site**		
	Hospital - NICU	5
	Hospital - Other inpatient	5
	Hospital - Outpatient	40
	Private Practice	83
	School	227
	Early Intervention	74
	Other	41
**Primary Age Group Working With**		
	Birth to 3	195
	Preschool (age 3-4)	308
	Early School (age 5-7)	297
	Late Elementary (age 8-10)	234
	Middle School (11-13)	134
	High School (14-18)	85
