# Supplementary material for: Pediatric Speech-Language Pathologists’ Use of Mobile Health Technology: Qualitative Questionnaire Study
Source: JMIR Rehabil Assist Technol. 2019 Sep 26;6(2):e13966. doi: 10.2196/13966 (PMC6787525; doi:10.2196/13966)
Supplement: Multimedia Appendix 3 [file rehab_v6i2e13966_app3.pdf]

Multimedia Appendix 3: Technology use by age group.

	≤ 35 years	>35 years	Total
**Do you use any technology in your clinical work?**			
**Yes, all of the time**	69	58	127
**Yes, some of the time**	135	104	239
**Rarely**	40	32	72
**Never**	10	7	17
	254	201	455
